# Fear, Shame, and Miscommunication: A Qualitative Study on Treatment Choice for Pelvic Organ Prolapse

**DOI:** 10.1007/s00192-026-06545-3

**Published:** 2026-03-06

**Authors:** Daphne Struik, Wendy Oosterbroek, Lisa R. van der Vaart, C. H. van der Vaart, Catharina J. van Oostveen, Astrid Vollebregt

**Affiliations:** 1 Vrije Universiteit Amsterdam, Amsterdam, The Netherlands; 2 Leiden University, Leids Universitair Medisch Centrum, Albinusdreef 2, 2333 ZA Leiden, The Netherlands; 3 Department of Obstetrics and Gynaecology, UMC, Amsterdam Reproduction & Development Research Institute, University of Amsterdam, Amsterdam, The Netherlands; 4 Department of Gynaecology, Bergman Clinics, Bilthoven, The Netherlands; 5 Spaarne Gasthuis Hospital, Spaarne Gasthuis Academy, Haarlem and Hoofddorp, Hoofddorp, The Netherlands; 6 Erasmus School of Health Policy & Management, Erasmus University Rotterdam, Rotterdam, The Netherlands; 7 Department of Obstetrics and Gynaecology, Spaarne Gasthuis, Hoofddorp, The Netherlands

**Keywords:** Facilitator–barrier study, Female pelvic floor dysfunction, Pelvic organ prolapse, Pessary, Shared decision-making, Surgery

## Abstract

**Introduction and Hypothesis:**

Pelvic organ prolapse (POP) negatively impacts the quality of life of many women. Effective treatment options include the use of a pessary and surgery. This study was aimed at exploring the facilitators and barriers influencing decision-making between pessary and surgical treatment from both the patients' and gynecologists’ perspectives.

**Methods:**

This qualitative study involved semi-structured interviews with 14 women from the own choice cohort in the PEOPLE study and 12 gynecologists from various hospitals throughout the Netherlands. Participants were purposively sampled to capture a range of experiences and professional backgrounds. The women varied in age and prolapse severity and had differing experiences with both pessary use and surgery, whereas gynecologists represented a mix of junior and senior specialists from academic and regional hospitals. Interviews were conducted until thematic saturation was reached. Data were analyzed using thematic analysis following Braun and Clarke.

**Results:**

Three main themes emerged. Treatment goals were a reflection of fears and misconceptions, illustrating the reasons behind women’s treatment choices; patient presentation delay as a result of ignorance and shame, addressing the societal taboos surrounding POP; and consultation at the gynecologist with communication gaps and decision-making challenges, highlighting the barriers faced during consultations with gynecologists.

**Conclusions:**

Optimal decision-making can be limited because of a lack of knowledge among the general population, patients, and general practitioners. Ignorance and feelings of fear and shame on the subject in patients further hinder the decision-making process. Addressing these barriers could improve the shared decision-making process and enhance patient satisfaction with their chosen treatment.

**Supplementary Information:**

The online version contains supplementary material available at 10.1007/s00192-026-06545-3

## Introduction

Pelvic organ prolapse (POP) is a prevalent condition in women that negatively influences their quality of life, impacting physical and emotional well-being [1]. In general, watchful waiting can be advised as primary treatment if the patient reports few to no symptoms. A moderate to severe symptomatic prolapse can be treated by either a pessary or surgery. Previous comparative studies between pessary and surgery for women with symptomatic POP show that both interventions reduce pelvic floor symptoms, although surgery is generally associated with better subjective improvement [2]. A recent non-inferiority trial, the PEOPLE study, showed that the use of a pessary was less effective than a surgical intervention when women rated the improvement in their symptoms [3, 4]. The PEOPLE study included both a randomized controlled trial (RCT) and cohort study arm, and subjective improvement of symptoms was consistently higher in the surgery group. Women dissatisfied with their initial treatment were free to quit or change therapies, and were followed up accordingly within the respective cohort. Interestingly, the RCT and cohort results differed in the percentage of women switching from pessary therapy to surgery. In the RCT pessary group, 54% of patients initially assigned to a pessary tried the pessary but ultimately underwent surgical treatment. In the pessary cohort group, 23.6% of patients who tried a pessary eventually opted for surgery [3, 4]. Although these results show that the use of a pessary and surgery are not equally effective in terms of symptom improvement, women who continued pessary treatment still reported a high satisfaction rate [3, 4]. The ease of use, low probability of complications, high satisfaction with continued pessary therapy, and its cost-effectiveness profile [5] still make pessary therapy a viable option for treatment [3, 4].

The remarkable difference in cross-over rate from pessary to surgery between women who were randomized compared with the own-choice cohort triggered us to explore which factors influence the treatment decision-making. In women with POP, treatment decisions are often influenced by factors including age, BMI, sex life, and severity of symptoms [4, 6]. Similarly, gynecologists consider the patients’ age and the specific type of prolapse when determining the most appropriate treatment approach, although they show a preference for the pessary as initial treatment [7]. However, most of this knowledge consists of quantitative research using predetermined factors, whereas the actual arguments may differ. Qualitative studies indicate that women's choices can be influenced by shame and prejudices due to a lack of knowledge, assumptions such as fear of complications from using mesh, and the belief that surgery permanently fixes POP [8].

As the fragmented literature shows evidence of several influential factors, but little argumentation for treatment choice, the need arises to draw a clear picture of the facilitators and barriers for patients and gynecologists that play a role in the decision-making process in current daily practice. Hence, this study is aimed at gaining an insight into the facilitators and barriers to satisfactory decision-making from both the patients’ and the gynecologists’ perspective. This information may consequently be used to help new patients to make the right treatment choice, thus preventing treatment crossovers, facilitating better outcomes and reducing costs.

## Materials and Methods

This study was designed and reported in accordance with the Consolidated Criteria for Reporting Qualitative research checklist. The completed checklist can be found in Appendix 1.

### Study Design

Following the PEOPLE RCT and cohort study, in which women expressed strong treatment preference and notable differences were observed in crossover rates between the RCT and the cohort, this qualitative study was initiated to determine the facilitators and barriers in the POP treatment decision-making process. To gain insight into perspectives in this matter, women with POP and gynecologists treating women with POP were included in this study. A general qualitative interview study based on interviews was used to provide the most valuable information, as participants would be able to express their experiences and thoughts in their own words without being restricted by a questionnaire [9].

To get insight into patient and health care provider beliefs considering the decision-making process, we used semi-structured interviews to minimize the impact of the researcher’s philosophies on the outcome and gather information on those topics that were most important for the participants [10].

### Participant Selection

#### Women with Pelvic Organ Prolapse

Eligible participants were women who participated in the “own choice” observational cohort of the PEOPLE study and completed the primary outcome questionnaire at 24-month follow-up, the Patient Global Impression of Improvement scale [11]. We decided to exclusively include women who participated in the observational cohort of the PEOPLE project as they had a strong a priori treatment preference and would be most representative of standard practice and the general population. When giving informed consent for the PEOPLE study, participants were asked for consent to be approached for follow-up research. We used a purposive sampling technique [12]; each fifth patient was selected from the list of patients in the cohort and contacted via email or letter. After providing consent, the women were approached by JR, RV, or DS through telephone calls. We aimed for maximum variation sampling and particularly aspired to equally include women from the pessary and surgery groups.

#### Gynecologists

In the selection of gynecologists, we wanted to acquire as many opinions as possible. Therefore, participants were selected from a variety of hospitals using purposeful maximum variation sampling [12]. Any gynecologist treating women suffering from POP was eligible, although we aimed for a variety in age, gender, location of practice, and experience of treating POP. Participants include gynecologists and subspecialized urogynecologists. Furthermore, we aimed to include some gynecologists who had previously participated in recruiting participants for the PEOPLE study, as well as gynecologists who had not been involved in the study. Participants were approached via email and informed consent was obtained.

### Study Population

#### Women with Pelvic Organ Prolapse

All women approached agreed to participate; no participants withdrew from the study. Data saturation was reached at 14 interviews, as no new codes or themes emerged [13]. The most common age categories were 60–69 and 70–79 years old. Six women received surgical intervention and 8 women initially started with pessary therapy. Of the women who started with pessary therapy, 3 switched to surgical intervention (Table 1).

**Table 1:** Participant characteristics: patients, obtained from the PEOPLE study

	Number
Age (years), range	49–81
40–49	1
50–59	4
60–69	4
70–79	4
80+	1
Surgery	5/14
Switch to surgery	3/9
POP severity before therapy	
Mild	3
Moderate	6
Serious	5
Improvement after therapy	
Much worse	2
No change	1
A little better	3
Much better	4
Very much better	4

*POP:* *pelvic organ prolapse*

#### Gynecologists

Sixteen out of the 20 contacted gynecologists responded to the email, stating their willingness to participate in the study. Data saturation was reached at 12 interviews. Of the 12 participants, 7 were female, 5 had been involved in the PEOPLE study, and 8 participants were specialized urogynecologists (Table 2). Participants’ most common age category was 40–49 years old, most had been in practice as a gynecologist for 10–19 years. The location of their practice was throughout the Netherlands and 9 participants worked at a teaching hospital.

**Table 2:** Participant characteristics: gynecologists

	Number
Age (years), range	(35–66)
30–39	2
40–49	5
50–59	3
60–69	2
Gender, male/female	5/7
Type of hospital	
General	1
Teaching	9
Academic	4
Years in practice	10 months to 28 years
0–9	3
10–19	5
20–29	4
Specialty in urogynecology	8

### Data Collection

The interview guide for women with POP was created in a meeting with the authors. After completing the PEOPLE project’s “own choice” cohort we wanted to elaborate on what facilitators and barriers to women with POP experience in the decision-making process lead to a certain treatment preference. Each interview started with a few short, demographically related questions (age, level of education, and duration of symptoms) for the patients, and for the gynecologists age, years in practice, and location of practice. After multiple open questions, specific questions on possible barriers and facilitators derived from previous research were included in the questionnaire [6]. The interview guide for the gynecologists was partially based on the topics that arose from the interviews with women with POP, to gain an insight into possible differences in the experience of the shared decision-making process. Additionally, after discussing previous research [7], we included further questions on the gynecologists’ treatment preferences and how they counsel their patients.

Both interview guides can be found in boxes 1 and 2; these contain condensed versions in English, as the original interview guides were in Dutch. The interview guides were revised and optimized when new information arose during the interviewing process, to ensure their understandability and completeness [14].

**Table:** 

Patients’ interview guide
• Why did you participate in the PEOPLE study? - Motivation for participation in the “free choice” cohort - Motivation for certain treatment selection • Knowledge of POP diagnosis and treatment • How do you feel looking back on your participation in this treatment? - Shared decision-making—treatment satisfaction • Did your time available for the study and treatment have an impact on your participation in the study and treatment of choice? • How did the people around you react to your participation in the study? Did they have any influence on you?

**Table:** 

Gynecologists’ interview guide
• How does the process unfold from the referral of a patient for pelvic organ prolapse complaints to treatment? - Preparation - Consultation - Follow-up • What prior knowledge do patients usually have? - Sources - Addressing prejudices • How do you inform the patient about the diagnosis and possible treatments for pelvic organ prolapse? - Supplementary materials, decision aids • How does the decision-making process for treatment choice go? How do you advise your patients through this process? - Patient’s choice - Treatment goals - Arguments for treatment • Do you observe a sense of shame among your patients regarding pelvic organ prolapse?

During the interviews, the interviewer maintained a neutral point of view to avoid leading questions and keep (non)verbal responses free from judgment and opinions [15].

Interviews with patients were carried out by JR (male), RV (female), and DS (female). After completing all interviews with patients, interviews with gynecologists were carried out by DS and WO(female). On average, interviews lasted 32 min. All interviews were carried out in Dutch, the native language of all participants and interviewers. For this paper, quotes and codes were translated^1^ into English using faithful translation.

Participants were interviewed either face-to-face, through video-calling, or via telephone call, depending on the participant’s preference and COVID-19 restrictions. Studies show that telephone interviews can be as reliable as face-to-face interviews [16]. Face-to-face interviews were performed at a location convenient for the interviewee. For example, in the clinic or at the patient’s house.

Interviewers had no prior relationship with participants.

### Data Analysis

Parallel to the data collection, data analysis was performed. All interviews were audio-taped and transcribed using verbatim transcription on Teams (version 1.6.00.33567) or transcribed manually. Recordings and auto-transcripts from Teams were deleted afterward. The interviews were coded using MaxQDA (version plus 2022) following the six-phase analytical process of Braun and Clarke [13]. First, two researchers (DS and WO) read all interview transcripts to become familiar with the data. All interviews were then open-coded to apply general codes to text fragments to clarify their origin. One researcher (DS) coded interviews with patients, another researcher (WO) coded interviews with gynecologists. Both students were supervised by CO during this process; in addition, CO conducted cross-checking of the codes to enhance the consistency and reliability of the analysis. Hence, a uniform coding system was used for all data. Third, code categories were determined. Of these categories, a mind map was constructed. Then, themes were conceptualized, multiple candidate themes were composed and reviewed by the complete research team (including DS, WO, CO, AV and LV) during multiple meetings. Last, after an analytic narrative was formed and themes were named, the interview transcripts were reviewed once more to verify whether these themes resembled the initial data. As Braun and Clarke describe an iterative process, some of these phases occurred simultaneously, providing the opportunity for continuous analysis of the process and for making adjustments where needed [13]. This way, interviews were conducted until no new codes and themes emerged and we regularly checked back with the raw data to ensure that the themes matched the participants’ experiences.

After defining the main themes, member checking was performed by presenting the results to a group of gynecologists independent of the study, to check for resonance with the people involved [17]. Feedback from participants was not obtained.

### Research Team

The research team consisted of several medical students completing their academic internships, namely JR, RV, DS, and WO. AV and HV are clinical urogynecologists and researchers, whereas LV was a PhD candidate in urogynecology at the time of the study. CO is a nursing dean and researcher with a post-academic degree and extensive experience in qualitative research.

### Ethical Considerations

This research was approved by the local review boards of the hospitals and institutions that participated in the study. The research was approved by the Medical Research Committee (MREC) Utrecht. The date of approval was 22 September 2015 and the METC protocol number is 14–533.

Data remained anonymous as participants’ names were converted into identification numbers. After completing this study, all data were stored for 10 years on a hard drive at the science department of the leading hospital.

## Results

### Overview of the Findings

After analysis of the findings, three main themes emerged. Within these themes, facilitators and barriers are described not as isolated factors but as motives that may either support or hinder the decision-making process. The themes are:

1. Treatment goals: a reflection of fears and misconceptions
2. Patient presentation delay: a result of ignorance and shame
3. Consultation with the gynecologist: communication gaps and decision-making challenges in treatment choice (Table 3)

The first theme describes women’s expectations of treatment for POP and arguments for treatment choice. Additionally, it provides insight into what information motivates women with POP to make their decision. The last two themes describe factors forming barriers in the treatment decision-making process for POP. These barriers include a lack of knowledge among women with POP and a lack of reliable information about the condition and its possible treatments, resulting in a delay in presentation. Additionally, during consultation, the lack of knowledge among women negatively influences their ability to obtain sufficient information to make a treatment decision. Subsequently, patients’ treatment goals are not fully attained because gynecologists may not always have a clear understanding of the considerations underlying patients’ preferences, leading to a communication gap and a more directive approach to treatment.

**Table 3:** Definitions of main themes

Theme	Definition
Treatment goals: a reflection of fears and misconceptions	Arguments for treatment preference were often based on patients own beliefs and (mis)conceptions rather than information provided by the gynecologist
	The power of story: women value experiences heard from others as an important source of information and reason for treatment preference
	Pursuit of a permanent solution: women have a strong desire for a permanent solution, which causes the least disturbance in their lives
	The barrier of shame: feelings of shame associated with discussing prolapse symptoms
Patient presentation delay: a result of ignorance and shame	Shame about prolapse symptoms results in limited access to information and a delayed patient presentation
	Difficulty talking about symptoms to others
	Thought they were the only ones with these symptoms
	Desire for more awareness
	Decrease in quality of life
	Gynecologists recognize misinformation
Consultation with the gynecologist: communication gaps and decision-making challenges in treatment choice	Women with POP would have preferred a better explanation of their complaints and possible treatment during consultation with the gynecologist, whereas gynecologists believe that their patients are sufficiently informed after consultation
	Difference in experience on treatment decisions made by the patient or gynecologist
	Use of decision aids
	Shortage of time

### Treatment Goals: A Reflection of Fears and Misconceptions

#### The Power of Story

Women with POP described various arguments to support their treatment preference. All motives for treatment preferences can be found in Table 4. Noticeably, these arguments were often based on their own beliefs and conceptions rather than information provided by the gynecologist. One of the most important motives appeared to be negative treatment experiences from others. If women were informed by acquaintances or relatives about a negative experience with a pessary or surgery, women tended to use this as an argument to prefer the contrary treatment.

Well because I—well there was a family member who used a pessary, they also had a prolapse and they did not like the pessary. Thus, I decided to choose the operation

(P7, surgery).

No, no I did not [read: consider trying a pessary]. I am quite radical, if I have any complaints, I want to do something about it, no temporary solutions

(P4, surgery).

**Table 4:** Motives for treatment preference

Motives if patients had no preference	Motives if patients preferred surgery over pessary	Motives if patients preferred pessary over surgery
I will take any treatment that is successful. I want to get rid of my complaints	The wish for a permanent solution	Comorbidities (coughing, walking with crutches, open heart surgery)
The doctor knows what is best for my body	Pessary is too much hassle	Scared of surgery
	Pessary has a big impact on your life. Surgery provides better freedom of movement	Scared of complications from surgery
	I am too young for all the hassle with a pessary	Heard negative stories about surgery in the Dutch media
	A pessary is not convenient when you become old and have dementia	Preference for conservative treatment first “saves another surgery”
	A pessary is not hygienic	Private home situation does not allow being admitted to the hospital
	They heard negative experiences from other women	

#### The Pursuit of a Permanent Solution

Frequently, women with POP expressed a strong desire for a definitive solution to their symptoms—ideally a one-time intervention after which the prolapse would no longer interfere with their lives. At the same time, they emphasized the importance of convenience and minimal disruption, favoring treatment options that involved the least “hassle” or burden. They wish for a practical solution with the smallest possible impact on their life.

Because I cannot do that my whole life, using that thing every day. I think that is a hassle. I just want to exercise and do everything that I normally do

(P9, surgery).

For some women, this resulted in a preference for surgery, as they did not want a treatment that required their own continuing input. Conversely, some women used the “no hassle” argument in preference for the pessary treatment, because planning a surgery, including recovery time, would have too much of an impact on their life.

With a pessary, you do not need to visit the hospital. You can just go on living your life

(P2, pessary, surgery).

Gynecologists acknowledge that their patients often base their treatment preference on factors other than the information that they provide them. For example, one gynecologist heard a woman recall that their grandmother had a pessary that irritated her skin, resulting in a lot of discharge and a noticeable smell. She therefore preferred not to use a pessary. Gynecologists also note that surgery has had a lot of negative media attention because of complications in mesh surgery.

That is very diverse as well. You hear people say ‘Yes, my neighbor has had surgery, which solved all her complaints,’ or ‘She had surgery, and suffered from many complications.’ Or ‘she has a ring and is able to do everything again’ or ‘She has a ring, and it fell out all the time. That is a disaster.’ It is very diverse; however, I must say that the experiences from their acquaintances are very often more influential than what I can sometimes tell them

(G10).

#### The Barrier of Shame

In most arguments for treatment preference, it appears that speaking and dealing with symptoms of POP are a source of shame. Women are ashamed of talking about prolapse issues; therefore, the few stories that they hear about others with POP determine their own beliefs. Moreover, women delay visiting a doctor, hoping that the symptoms will resolve without an intervention. Women experience a taboo in dealing with medical issues concerning their private parts, and one participant even reported feeling a taboo around touching her own genitals. Consequently, many expressed a desire for their complaints to be resolved as quickly as possible, preferring a treatment that offers a one-time solution with minimal hassle.

That place [read: the vagina] is taboo for your fingers, for my fingers. That is how I experience it. I cannot touch myself there. And you will be forced to take the pessary out and put it back in yourself

(P5, pessary).

### Patient Presentation Delay: A Result of Ignorance and Shame

Most women with POP experienced symptoms for years before consulting their doctor. Many reported feeling ashamed about physical complaints concerning their genitals and highlighted the societal taboo around discussing these issues. This made it difficult for them to talk about their symptoms with friends, family, health care professionals, or even their partner. As a result, women often had limited access to information about prolapse symptoms and potential treatment options.

Well... How long... There is a certain moment when you realize something is sticking out down there, that should not be there. I cannot tell you how long I already had complaints before that moment, because you have these symptoms, however you cannot describe exactly what it is. And you are embarrassed about where these complaints are located. At some point, you decide to consult a doctor, but how long I waited... You do not even tell your partner about it

(P5, pessary).

Many women reported being unaware that their complaints were common, describing their experience as feeling that they were the only ones living with these symptoms.

I had the feeling I was the only one experiencing this

(P6, surgery).

On the rare occasion when women did start a conversation about their pelvic floor disorder with acquaintances or relatives, it appeared that other women suffered from similar symptoms. Hearing about similar experiences from other women made them feel relieved. Hence, women stressed the importance of awareness regarding prolapse symptoms, the diagnosis of POP, and possible treatment options among the general population.

But I did have the idea that women do not usually want to talk about having prolapse symptoms. But I only experienced that later.... I also noticed the taboo on the subject. I only heard about it from other women when I started to talk about it myself. Then I learned how many women suffer from this and how many underwent treatment for it

(P6, surgery).

Various women with POP denied and avoided their complaints, until these became untenable and consulting a doctor was inevitable. Most women explained that they decided to consult the doctor because of a decrease in their quality of life, provoked by the progression of their symptoms. This included symptoms interfering with their daily life, (a fear of) incontinence, and the need to manually push back the protrusion before being able to defecate. Women reported that they had to overcome the shame to regain a normal quality of life.

Prior to the operation I was scared to leave the house. I was scared I had to use the restroom but was not able to find one. That is the reason I decided something needed to happen. It is no normal life if you must stay inside day and night even before you are 70 years old, because you fear you need to use the restroom

(P2, pessary, surgery).

Gynecologists believe that people hardly ever encounter the topic of POP in day-to-day life. Although incontinence may be mentioned among acquaintances, POP remains undiscussed.

Gynecologists mention that women suffering from the condition do not recognize their symptoms and that there is a lack of reliable information on POP symptoms and treatment for women with POP. Some gynecologists assume that women think that it is a normal change for their aging bodies that they just have to deal with. This is mentioned as one of the reasons why some women suffer from POP for far longer than necessary.

But sometimes I still see patients who say: ‘Well, I’ve had this for years and I thought, well, it comes with aging.’ And weren’t aware anything could be done about it

(G12).

I think it is embarrassment after all. These are your intimate parts; you do not easily talk about them. Periods are different, you can talk about that. But about this, this is too personal. I think everyone will agree

(P10, pessary).

Gynecologists mention that they often spend time rectifying women’s unfounded prejudices about the treatment options.

If they’d heard positive experiences, then they thought, or heard from the general practitioner or from friends, well, then they wanted to have the same treatment their friend had had as well

(G3).

In addition to misinformation from acquaintances or from the internet, information provided by some general practitioners (GPs) can also be a source of confusion when consulting a gynecologist. Women reported that GPs often did not elaborate on the possible POP treatments. Additionally, gynecologists have experiences with GPs who attribute symptoms to the prolapse that are not actually related, tell the patient that they will need surgery because a pessary will not work, or explain that the uterus is the problem, making the patient think that they will need a hysterectomy.

It often occurs because the general practitioner didn’t state the right diagnosis (...) and then we’ll have to go way back because the diagnosis isn’t right. And then the whole plan changes and you’ll notice confusion (in the patient), that is often difficult

(G6).

Some GPs are knowledgeable about POP and start appropriate treatment; they prescribe physiotherapy or place a pessary. However, misunderstandings may occur when they eventually refer their patients to a gynecologist. For example, when one pessary has failed, some women think that pessary treatment is no longer an option and that they will definitely need surgery.

In general, gynecologists would like to see an optimized care process, where women with POP receive appropriate care from knowledgeable GPs first before being referred to the second line of care, the gynecologist. This will prevent confusion and simplify the decision-making process for women with POP.

### Consultation with the Gynecologist: Communication Gaps and Decision-Making Challenges in Treatment Choice

Women with POP reported that they hear, understand, and remember less information than the gynecologists provide during consultation. Various participants stated that they were unable to recall important information, such as risks and side effects. Women mention being too overwhelmed by emotions to be able to hear and understand all the information. They would have preferred a better explanation of all treatment options and more time to consider this new information.

Uhm, yes probably [the doctor explained about risk and side-effects]. Yes, I assume they absolutely discussed that. However, I cannot recall it

(P14, pessary).

When it is time to decide on a treatment, women mention that they do not feel entirely free to choose whatever they want.

I think the doctor was not bad at all, however, I did not have the autonomy to choose my own treatment, and he did not try to make that easier for me

(P3, pessary).

In contrast to the beliefs of women with POP, gynecologists have confidence that their patients are sufficiently informed after their consultation and emphasize that patients always have the final choice regarding their treatment. They apply multiple methods to fully inform the women with POP: verbal explanations, pictures, written information, and websites with videos. However, they realize that not all women will be able to retain all the information provided during their consultation. Hence, some do not explain everything fully and refer their patients to written information or a website instead.

Of course, you know in advance that a large part of the information you provide the patient with doesn’t stick well. But I think that, if I provide additional information, they are truly better informed

(G11). Interviewer:

And will you leave the final choice for the treatment to the patient?

G5:

Yes, yes. That is in principle always the case.

Some gynecologists admit that they have an opinion on what would be best for their patients. They may prefer to always try a pessary first and present it in a way that encourages their patients to agree with the proposed plan. G5:

I always guide [read: the patient] toward a pessary.

Interviewer:

In everyone?

G5:

Yes, unless physical examination shows that it is a bad idea.

Moreover, pessaries are regularly already inserted during the physical examination to determine the characteristics of the prolapse, so women with POP need to accept or decline this option directly at their first visit to the gynecologist.

My opinion, simply speaking, is that you can always try a pessary first, because nothing ventured, nothing gained. It is a very easy way to solve a prolapse

(G8).

According to gynecologists, some women are easily persuadable, older women in particular, among whom the taboo is more present. Younger patients can advocate for their opinion and disregard the gynecologist’s counseling.

Of course, tailored to the level of the patient I try to correct it. Especially when I think that the patient bases her choice on incorrect information, I am sooner inclined to say ‘Well, reread it [read: the information] again, watch this video and I’ll call you in two weeks on what we’ll decide to do.’

(G11).

Some gynecologists mention that the use of decision aids (DAs) could help in decision-making. However, many of the interviewed gynecologists mention hardly using them, claiming that they are not well adapted to the situation of the patient, too expensive for daily use, or simply not contributory, as the gynecologist can explain the treatment options equally well.

To be honest, well, because I tend to be quite conservative, I wonder what a decision aid would exactly contribute to me or to the patient

(G4).

Well, I personally think, yes, in my opinion decision aids are mostly fun. I enjoy informing patients very well and having them take control of their own care. I mostly like decision aids in concept, but one way or the other I don’t think all of them work very well. However, I do think they are good at describing the pros and cons

(G1).

A barrier in decision-making is the shortage of time for consultation. Some gynecologists mention feeling rushed to finish within the scheduled time frame; they would be able to put more energy into informing patients if there were time to do so. Additionally, women do not get enough time to have all their questions answered. Factoring in the taboo, which contributes to women feeling uncomfortable asking all their questions, women end up feeling inadequately informed and dissatisfied.

Gynecologists illustrate that a lot of information needs to be explained. Consequently, to correct the misconceptions of women with POP as well as fully to inform them, more time is required.

## Discussion

This study explored the facilitators and barriers that women with POP and gynecologists encounter in the treatment decision-making process for POP. Although other studies may compare patient characteristics with treatment preferences or outcomes, this qualitative study focuses on the reasoning behind treatment decision-making and common contributing factors.

### Main Findings

The findings of this qualitative study revealed three themes illustrating the barriers encountered by patients and gynecologists in treatment choice for POP, as described in the section Overview of the Findings. Although the study explored both barriers and facilitators, participants predominantly addressed barriers; consequently, mostly barriers are represented in the reported themes.

The first theme illustrates that women’s treatment goals were largely shaped by fears and misconceptions, often influenced by the negative experiences of others or misinformation from general practitioners (GPs) and the internet, rather than evidence-based medical advice. The second theme shows that many women delayed seeking treatment owing to ignorance and societal taboos around discussing genital health, further inhibiting their access to information, contributing to a lack of knowledge about their condition. Additionally, our findings suggest that there might be gaps in GPs’ knowledge about POP. According to patients and gynecologists, many GPs may not always have sufficient experience or information to fully inform, counsel, or treat patients. This could contribute to limited knowledge about POP among women during their consultations with gynecologists.

The last theme concludes that fears and misconceptions among women, combined with communication gaps during consultations, led to difficulties in making informed decisions. Women explain that they feel inadequately informed or overwhelmed. However, as women base their decisions on misconceptions, they are likely to feel uninformed and dissatisfied with their treatment of choice.

To date, limited research has been conducted on women’s motivation regarding treatment choice and the factors influencing it. Most studies use a quantitative approach, and are thus bound to predetermined arguments. Our study combines the perspectives of women and gynecologists in their own words, thus contributing novel insights into the factors influencing decision-making. Previous studies concur that the general population is largely unaware of what POP entails, leading to women being unable to identify the cause of their symptoms [18]. This results in a delayed presentation and treatment decisions based on misconceptions, causing treatment dissatisfaction. Women in our study stress the need to be well-informed on their treatment options and throughout the decision-making process. The wish for an active role in decision-making has been researched by Sung et al. They concluded that 44% of the respondents preferred an active role, 47% wished for a collaboration with their doctor, and 9% desired a passive role [19]. This is supported by our participants, who all stressed the importance of the inclusion of patients in the decision-making process. These findings are further supported by the PEOPLE study, which demonstrated a lower treatment crossover rate in the cohort group compared with the RCT group, implying greater patient satisfaction when treatment choice is patient-driven rather than randomly assigned [3, 4]. Basu et al. found that the success rates of treatment were the most important factor in the decision-making process for prolapse treatment [8]. However, women in our study were more likely to base their treatment preferences on prejudices and the experiences of others.

Our results further revealed that women found that the information on POP provided by GPs to be lacking. The literature does not specify the extent of GPs’ knowledge regarding POP. However, van Leeuwen et al. describe that 10 years post-graduation, a GP’s knowledge diminishes and shifts to focus more on chronic diseases [20]. However, our study did not include an assessment of GPs’ knowledge; therefore, we cannot draw conclusions about their expertise in this area based on our results.

In the Netherlands, there are guidelines concerning POP for gynecologists, which are also accessible to GPs [21, 22]. There are no guidelines specifically for the first line of care. These POP guidelines, originating from 2014, are slightly outdated. They elaborate extensively on urodynamic investigation, possible additional research, and potential surgical treatments. However, they lack comprehensive information on possible misconceptions to consider when informing and treating women with POP [21]. Other international guidelines, such as the NHS guideline for POP, are more oriented toward GPs. They include a brief pathway guide with options for primary care, reasons to refer to a urogynecologist, and a link to a patient information website. However, these guidelines do not elaborate on possible misconceptions either [23].

A possible method of informing women about their condition and treatment options is by using DAs. A DA is an online resource, accessible to individuals with basic computer skills, that supports medical decision-making.

In the Netherlands, several DAs on POP are available for use by gynecologists or GPs. Most DAs provide general information and are not patient-specific [24, 25]. They offer objective information on treatment options, including the likelihood of success and the risks of side effects. However, they lack information to dispel possible misconceptions, which this study identifies as the most significant factor influencing treatment preferences. Our findings also showed that patients often sought medical help only when their symptoms began to significantly affect their quality of life. Women frequently mentioned issues such as sexual functioning and co-existing incontinence as key factors prompting them to seek treatment (Theme 2). These aspects appear to play an important role in the decision-making process and could be addressed more explicitly within DAs. Including such patient-reported factors may help to ensure that DAs better reflect the real-life experiences and motivations of women with POP [24]. Furthermore, although some DAs use clear language, not all are easy to use and understand [24, 25].

### Strengths and Limitations

The strength of this study is the inclusion of a diverse range of women and gynecologists, which allows for the exploration of both perspectives on this topic. This approach provides a comprehensive view of the entire decision-making process in POP and offers new insights into the arguments and considerations that patients use when making treatment decisions.

A few limitations should be considered when interpreting the findings. First, interviews were conducted by various interviewers, which may have led to some inter-interviewer variability [26]. However, as all interviewers had to adhere to the interview guide and were all guided by CO (an experienced interviewer), the impact of this variation was minimized. Moreover, through the involvement of multiple interviewers, the influence of any single interviewer on the overall findings was reduced, thereby decreasing the risk of systematic interviewer bias [27]. Second, for this study, we were interested in women who were reluctant to participate in the RCT of the PEOPLE project. Therefore, these findings may not be representative of the general population, as these participants had a strong treatment preference. Third, each interview set was coded by one researcher, which may limit inter-coder reliability. However, analytical rigor was maintained through cross-checking of the codes, a shared coding framework, supervision by senior researchers, documentation of analytical decisions, and team discussions of emerging themes. The credibility of the findings was further supported through member checking with independent gynecologists. Last, because this study was performed in the Netherlands, the results may be less generalizable in countries with different health care systems.

### Recommendations

These study findings suggest several key recommendations to address the stigma and knowledge gaps surrounding POP and to improve access to effective treatment. First, increasing public awareness can help to reduce taboos and encourage more open discussions, making it easier for individuals to seek help. Educating the general population, not just affected individuals, is essential for dispelling myths and fostering a more supportive environment [28]. As GPs are often the initial point of contact for women in the health care system in the Netherlands and can provide valuable conservative treatment, it is recommended to improve the pathway to treatment, starting with an updated guideline and enhanced training for GPs in diagnostics and first-line treatment. Additionally, further research could investigate possible facilitators and barriers in POP treatment among GPs. Last, the use of improved DAs by gynecologists and GPs can ensure that women with POP are informed of all treatment options, empowering them to make decisions aligned with their needs [29]. Improved patient information and the use of DAs may also help to address the common challenge of limited consultation time faced by many gynecologists.

## Conclusion

Interviews with gynecologists and women with POP revealed that optimal decision-making can be hindered by a lack of knowledge among the general population, patients, and GPs, compounded by ignorance, fear, and shame experienced by women. Misalignment in communication between the patient and gynecologist results in women feeling insufficiently informed and not fully capable of making an informed choice. Moreover, gynecologists should be aware that women often base their treatment decisions on their own beliefs and misconceptions rather than the information provided by health care professionals. Increasing awareness in the general population and normalizing the condition would be the first step toward removing its taboo and improving the decision-making process for women with POP. Combined with the optimization of first-line care and the implementation of improved DAs, the patient decision-making process and treatment satisfaction may be enhanced.

## Supplementary Information

Below is the link to the electronic supplementary material.

Supplementary file1 (PDF 369 KB)

## Data Availability

The original Dutch quotes used in this study are available from WO or DS, upon reasonable request.

## Footnotes

1 All interviews and coding were initially performed in Dutch. Translations followed a faithful approach, capturing the intended meaning in as many of the original words as possible, while improving grammatical correctness and clarity. This way, the word-for-word translation of this quote: Because I that not my whole life can do, every day with such thing. That find I hassle. would turn into this: Because I cannot do that my whole life, using that thing every day. I think that is a hassle. All final translations were compared with the original Dutch text afterward to ensure that the translation was true to the original meaning.
